# Construction of Monophosphoryl Lipid A Producing *Escherichia coli* Mutants and Comparison of Immuno-Stimulatory Activities of Their Lipopolysaccharides

**DOI:** 10.3390/md11020363

**Published:** 2013-01-31

**Authors:** Yaning Han, Ye Li, Jiuzhou Chen, Yanzhen Tan, Feng Guan, Xiaoyuan Wang

**Affiliations:** 1 State Key Laboratory of Food Science and Technology, Jiangnan University, Wuxi 214122, China; E-Mails: ya-ya163@163.com (Y.H.); liye@jiangnan.edu.cn (Y.L.); jiuzhou1103@hotmail.com (J.C.); yanzhentan@163.com (Y.T.); 2 Key Laboratory of Carbohydrate Chemistry and Biotechnology of Ministry of Education, School of Biotechnology, Jiangnan University, Wuxi 214122, China; E-Mail: fengguan@jiangnan.edu.cn

**Keywords:** lipopolysaccharide, lipid A, MPLA, endotoxin, vaccineadjuvant, *Escherichia coli*, murine macrophage

## Abstract

The lipid A moiety of *Escherichia coli* lipopolysaccharide is a hexaacylated disaccharide of glucosamine phosphorylated at the 1- and 4′-positions. It can be recognized by the TLR4/MD-2 complex of mammalian immune cells, leading to release of proinflammatory cytokines. The toxicity of lipid A depends on its structure. In this study, two *E. coli* mutants, HW001 and HW002, were constructed by deleting or integrating key genes related to lipid A biosynthesis in the chromosome of *E. coli* W3110. HW001 was constructed by deleting *lacI* and replacing *lacZ* with the *Francisella novicida* *lpxE* gene in the chromosome and only synthesizes monophosphoryl lipid A. HW002 was constructed by deleting *lpxM* in HW001 and synthesizes only the pentaacylated monophosphoryl lipid A. The structures of lipid A made in HW001 and HW002 were confirmed by thin layer chromatography and electrospray ionization mass spectrometry. HW001 and HW002 grew as well as the wild-type W3110. LPS purified from HW001 or HW002 was used to stimulate murine macrophage RAW264.7 cells, and less TNF-α were released. This study provides a feasible way to produce interesting lipid A species in *E. coli*.

## 1. Introduction

Lipopolysaccharide (LPS), known as endotoxin, is the prominent constituent in the outer leaflet of outer membranes in most Gram-negative bacteria [1,2,3] and plays an important role in membrane permeability, cell adhesion and stability [4]. It can be divided into three parts: lipid A, core-oligosaccharide [5] and *O*-antigen repeats. Lipid A, the hydrophobic anchor of LPS, is responsible for the bioactivity of LPS [6]. It can be recognized by the TLR4/MD-2 complex of mammalian immune cells, leading to release of cytokines, such as tumor necrosis factor-α (TNF-α) and interleukin-6 (IL-6), upregulation of MHC class II molecules on antigen-presenting cells [6,7] and generation of strong Th1 responses [8].

**Figure 1:**
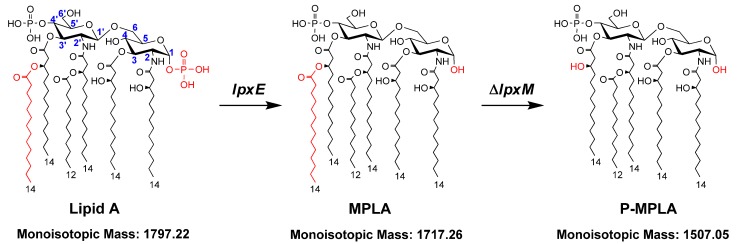
The strategic diagram of structural modification of *E. coli* lipid A. The numbers that specify the glucosamine ring positions and the fatty acid chain length of lipid A are indicated. The gene *lpxE* encodes LpxE, which removes the phosphate group from the 1-position of lipid A. The gene *lpxM* encodes LpxM, which adds a secondary tetradecanoyl residue at 3′-position of lipid A. Therefore, expression of *lpxE* in *E. coli* changes the structure of lipid A to monophosphoryl lipid A (MPLA); expression of *lpxE* and deletion of *lpxM* in *E. coli* changes the structure of lipid A to pentaacylated MPLA (P-MPLA).

Kusumoto’s group has synthesized lipid A molecules with different structures and demonstrated that the toxicity of lipid A is closely related to its precise chemical structure [9]. Small amounts of lipid A with the right chemical structure is beneficial for the host to clear the invading microbe; high levels of lipid A, however, lead to the release of high levels of circulating cytokines, whose procoagulant activity could damage the microvasculature and facilitate the syndrome of septic shock [10]. Wild-type *Escherichia coli* lipid A (Figure 1) contains two phosphate groups and six acyl chains [1,2] and activates both TLR4–TRAM–TRIF and TLR4–Mal–MyD88 signaling pathways [11,12]. Lipid A containing only one phosphate group weakens the ligand affinity, induces structural rearrangement of the TLR4/MD-2 complex [11] and selectively activates the TLR4–TRAM–TRIF signaling pathway, leading to secretion of lower levels of cytokines [13]. This suggests that the monophosphoryl lipid A (MPLA) is much less toxic than the wild-type lipid A, but still retains the immunomodulatory properties. In addition, *Salmonella typhimurium* lipid A lacking a secondary acyl chain at the 3′-position, that is, the pentaacylated lipid A, is also less toxic than the wild-type lipid A [14].

Since MPLA and pentaacylated lipid A are immunogenic, but less toxic [15], they could be used as vaccine adjuvant to enhance the strength and duration of the immune response to antigens [8,16]. However, most Gram-negative bacteria, such as *E. coli*, synthesize lipid A with two phosphate groups and six acyl chains. In recent years, the biosynthesis of lipid A in *E. coli* has been well characterized, and several enzymes that modify the structure of lipid A are reported. Therefore, now it is possible to construct *E. coli* strains to synthesize lipid A with the desired structures. For example, deleting the gene *lpxM* in *E. coli* can make it synthesize pentaacylated lipid A [17]. Expressing *Francisella novicida lpxE* in *E. coli* can remove the phosphate group from the 1-position of lipid A [18,19] (Figure 1).

In this study, *E. coli* mutants that synthesize hexaacylated MPLA or pentaacylated MPLA (P-MPLA) were constructed by integrating and/or deleting key genes related to lipid A biosynthesis and modification in the chromosome. The structures of lipid A in these mutants were confirmed by thin layer chromatography (TLC) and electrospray ionization mass spectrometry (ESI/MS). All mutants grew as well as the wild-type, and their LPS induces less TNF-α than the wild-type *E. coli* LPS when used to stimulate murine macrophage RAW264.7 cells. This study provides a convenient method to produce novel lipid A vaccine adjuvant in *E. coli*.

## 2. Results and Discussion

### 2.1. Construction of *E. coli* Strains that Can Synthesize MPLA or P-MPLA by Chromosomal Gene Integration and/or Gene Deletion

**Figure 2:**
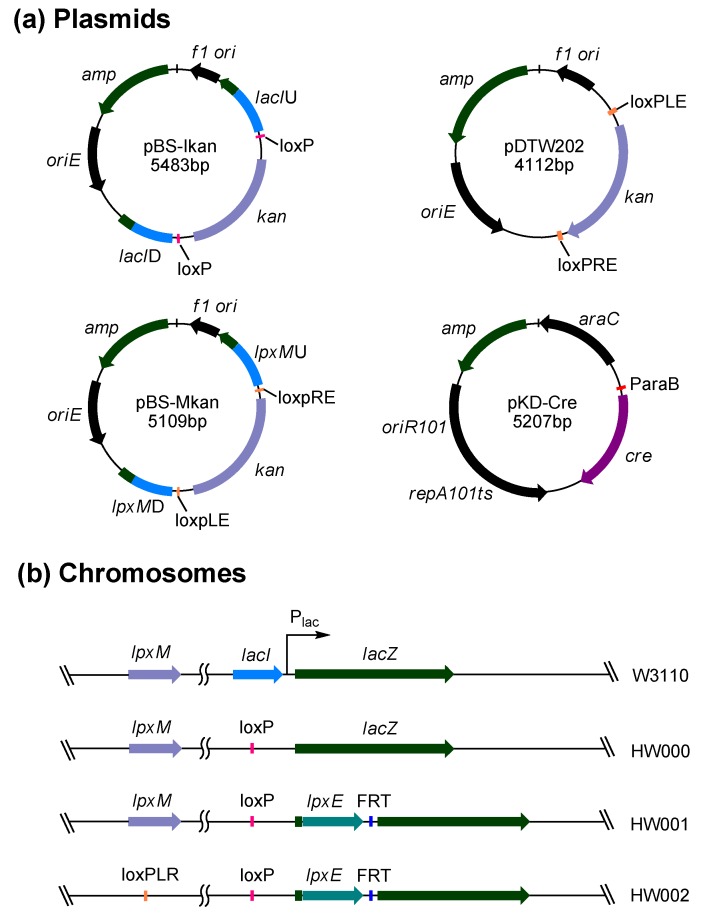
Plasmids and *E. coli* mutant strains constructed in this study. (**a**) Maps of four plasmids pBS-Ikan, pDTW202, pBS-Mkan and pKD-Cre constructed in this study; (**b**) The comparison of chromosomes of *E. coli* strains W3110, HW000, HW001 and HW002.

Four plasmids, pBS-Ikan, pDTW202, pBS-Mkan and pKD-Cre, were constructed to facilitate the chromosomal gene integration and/or gene deletion in *E. coli* (Figure 2a). Plasmid pBS-Ikan contains the fragment *lacI*U-loxp-*kan*-loxp-*lacI*D, which was designed to facilitate the deletion of the *lacI* gene and the subsequent removal of the inserted *kan* gene from the chromosome. Plasmid pDTW202 contains the fragment loxpLE-*kan*-loxpRE, which was designed for the easy removal of the *kan* gene by recombination of loxpLE and loxpRE. Plasmid pBS-Mkan contains the fragment *lpxM*U-loxpLE-*kan*-loxpRE-*lpxM*D, which was designed to facilitate the deletion of the *lpxM* gene and the subsequent removal of the *kan* gene from the chromosome. Plasmid pKD-Cre was designed to remove the inserted *kan* gene from the chromosome at the later stage of deletion.

Three *E. coli* mutants, HW000, HW001 and HW002, were constructed by using the above plasmids (Figure 2b). HW000 was constructed by deleting the *lacI* gene to avoid the lactose regulation. HW001 was constructed by integrating the *F. novicida lpxE* into the site of *lacZ* gene in the *lac* operon of *E. coli* HW000. HW002 was constructed by deleting the *lpxM* gene in the chromosome of HW001. The correct insertion or deletion of these genes and the removal of *kan* in these three *E. coli* mutants were confirmed by PCR analysis and DNA sequencing.

**Figure 3:**
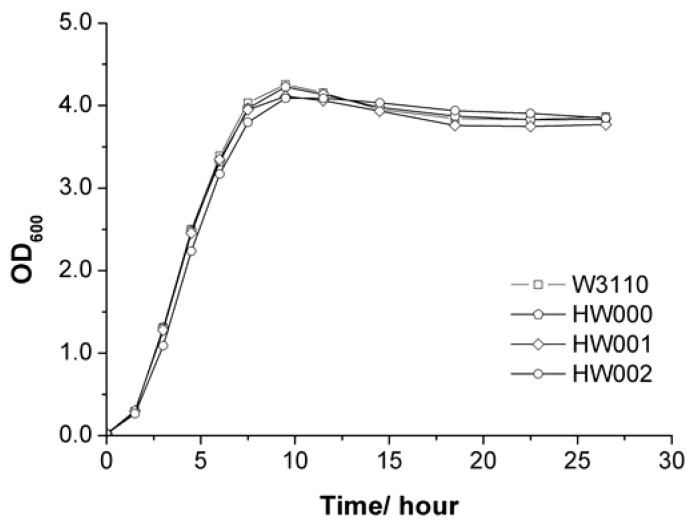
Comparison of growth of *E. coli* strains W3110, HW000, HW001 and HW002.

The growth rates of all these *E. coli* mutants were similar to that of wild-type W3110 (Figure 3). 

### 2.2. Structure Analysis of Lipid A Extracted from Three *E. coli* Mutants

**Figure 4:**
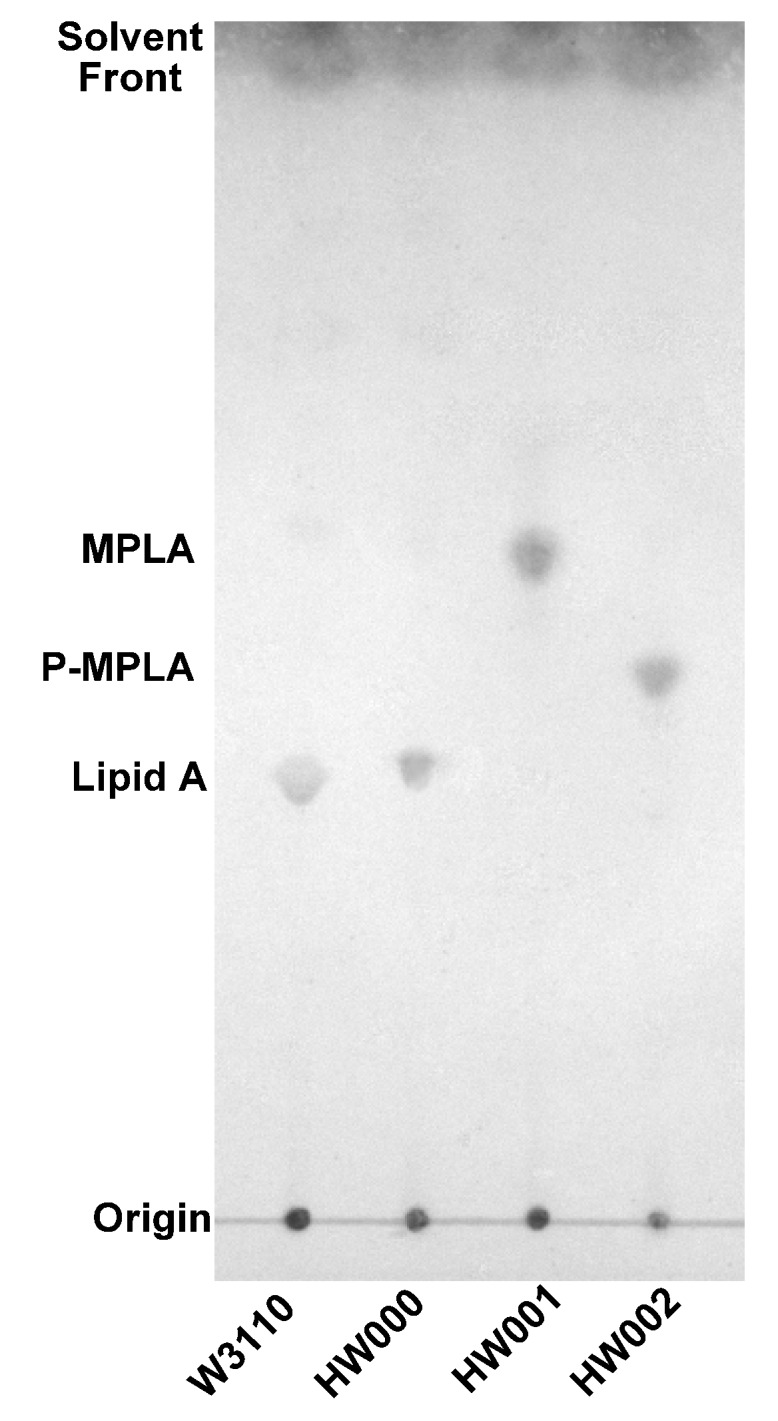
TLC analysis of lipid A isolated from *E. coli* strains W3110, HW000, HW001 and HW002. The lipid A samples were dissolved in chloroform/methanol (4:1, v/v), spotted onto a silica gel 60 TLC plate and developed in a solvent containing chloroform, methanol, water and ammonia (40:25:4:2, v/v/v/v). The plate was dried and sprayed with 10% sulfuric acid in ethanol and the positions of lipid A on the plate were visualized by charring at 175°C.

Lipid A was isolated from strains of W3110, HW000, HW001 and HW002 and analyzed by TLC (Figure 4). The typical lipid A band was shown on the TLC for samples isolated from W3110 and HW000. Considering the hydrophilic property of phosphate and hydrophobic property of fatty acid chain, lipid A containing less phosphate groups and more and longer fatty acid chains should migrate faster on TLC. A more rapidly migrating substance was observed on the TLC in the sample from HW001; likely MPLA. For the sample from HW002, a substance migrating faster than lipid A, but slower than MPLA, was observed; likely P-MPLA. 

**Figure 5:**
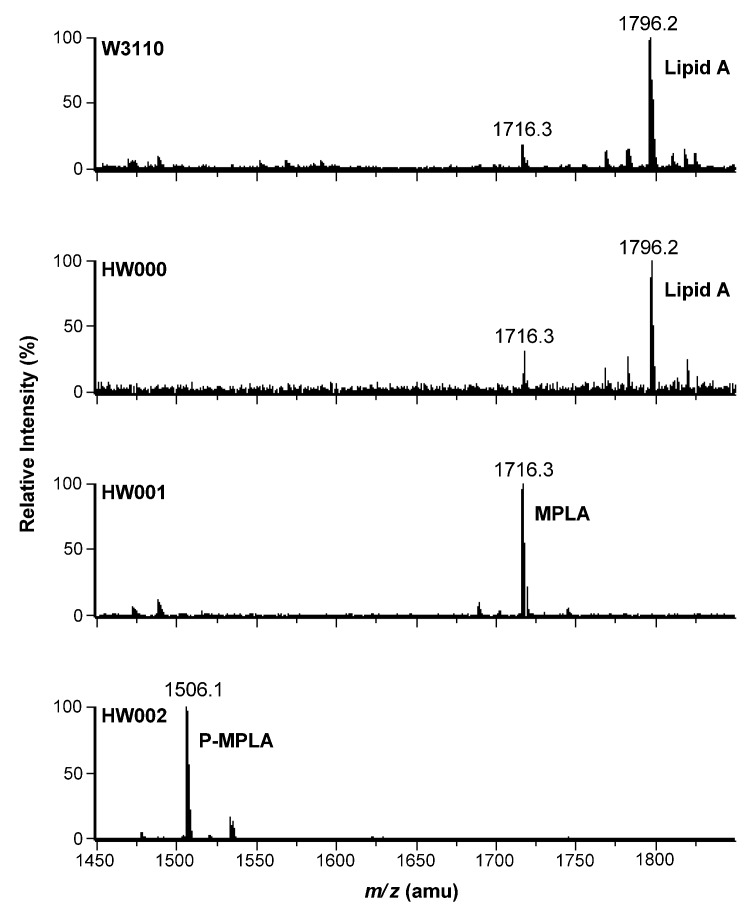
ESI/MS analysis of lipid A isolated from *E. coli* W3110, HW000, HW001 and HW002. Lipid A samples were dissolved in chloroform/methanol (4:1, v/v) and subjected to ESI/MS in the negative ion mode.

Lipid A isolated from W3110, HW000, HW001 and HW002 were further analyzed by ESI/MS (Figure 5). The lipid A isolated from W3110 yielded a major peak at *m/z* 1796.2 in the spectrum, suggesting that the peak is created by the molecular ion [M − H]^−^ of lipid A. The minor peak at *m/z* 1716.3 might arise by loss of a phosphate, indicating that the hydrolysis conditions for the cleavage of the lipid A from the core-oligosaccharide was too harsh and resulted in a partial loss of the α-anomeric C-1-phosphate at GlcN(I). Lipid A isolated from HW000 showed a similar pattern as that from W3110. In the spectrum of lipid A isolated from HW001, there is only one major peak at *m/z* 1716.3, suggesting that the lipid A in HW001 is completely dephosphorylated at the 1-position. In the spectrum of lipid A isolated from HW002, there is only one major peak at *m/z* 1506.1, suggesting that lipid A isolated from HW002 is P-MPLA. Both TLC and ESI/MS data indicate that HW001 can convert all lipid A into MPLA and HW002 can convert all lipid A into P-MPLA. This indicates that the gene *lpxE* inserted in the chromosome of HW001 and HW002 was well expressed and LpxE can modify the structure of lipid A *in vivo* and that *lpxM* encodes the only late acyltransferase in *E. coli* that adds a secondary acyl chain to the 3′-position of lipid A.

### 2.3. Comparison of the Stimulation Activities of LPS Isolated from Different *E. coli* Mutants

**Figure 6:**
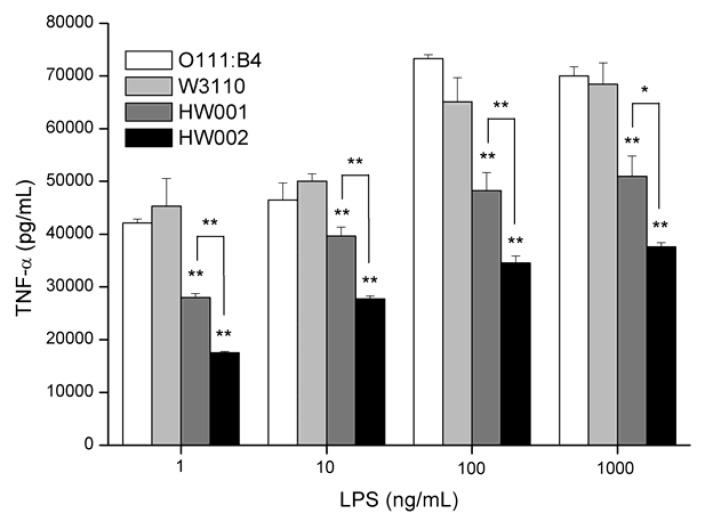
Cytokine concentrations in tissue-culture supernatants of RAW264.7 cells stimulated by increasing concentrations of lipopolysaccharide (LPS) sample. Supernatants from RAW264.7 murine macrophages were collected after 24 h stimulation with LPS and tested for the presence of TNF-α. One-way analysis of variance was used to evaluate differences in cytokine concentrations. Significant differences between stimulations by LPS from different sources were shown. * *p* <0.05, ** *p* < 0.01. The experiments were performed in triplicate, and data were shown as means ± standard deviation.

LPS was purified from W3110, HW001 and HW002 and used to stimulate macrophage cell line RAW264.7. Levels of TNF-α released by the cells after stimulation were examined (Figure 6). *E. coli* O111:B4 LPS was used as a positive control, and PBS (phosphate-buffered saline) was used as a negative control.

The levels of TNF-α released by RAW264.7 cells were proportional to the concentration of LPS up to 100 ng/mL, but there was little difference between LPS concentrations of 100 ng/mL and 1000 ng/mL. At all concentrations, lower levels of TNF-α were released when RAW264.7 cells were stimulated by HW001 LPS and HW002 LPS than by the controls O111:B4 LPS or W3110 LPS (Figure 6). The lowest levels of TNF-α were induced by HW002 LPS from 17,472 pg/mL at 1 ng/mL to 37,578 pg/mL at 1000 ng/mL, which were 39% and 55% of the levels induced by W3110 LPS, respectively. 

These results demonstrated that LPS from HW001 and HW002 induced lower secretion of TNF-α than that of wild-type *E. coli* LPS, suggesting they are good candidates for vaccine adjuvant development. The changes in the number of the acyl chains and the phosphate groups of lipid A might decrease the binding ability by TLR4/MD-2 and lead to the lower release of TNF-α.

## 3. Experimental Section

### 3.1. DNA Preparation and PCR Techniques

Restriction enzymes, shrimp alkaline phosphatase, T4 DNA ligase and DNA ladder were purchased from Sangon (Shanghai, China). Plasmid DNA was prepared by using the EZ-10 spin column plasmid mini-preps kit from Bio Basic Inc. (Markham, Canada). PCR reaction mixtures (50 μL) used in this study contain 5 μL 10× Ex Taq buffer, 4 μL dNTP mixture (2.5 mM each), 1 μL plasmid template (100 ng/μL), 1 μL forward primer (20 μM), 1 μL reverse primer (20 μM) and 0.5 μL TaKaRa Ex Taq DNA polymerase. The PCR reaction was first heated to 94 °C and maintained for 10 min, followed by 35 cycles of denaturation (94 °C for 30 s), annealing (30 s at 55 °C or 58 °C) and extension (72 °C for 3 min). At the end, an additional 10 min incubation at 72 °C was used. PCR products were purified by using the TIANgel midi purification kit from Tiangen (Beijing, China). Primer synthesis and DNA sequencing were performed by Sangon. The sequences of primers used in this study are listed in Table 1.

**Table 1:** Primers used in this study. The recognition sites for restriction enzymes are underlined.

Primers	Sequences (5′→3′)	Restriction site
*lacI*U-F	CCGCTCGAGACAGTATCGGCCTCAGGAAGAT	*Xho*I
*lacI*U-R	CGGAATTCTTAATGCAGCTGGCACGACAGG	*EcoR*I
*lacI*D-F	GAAGATCTGCGACATCGTATAACGTTACTG	*Bgl*II
*lacI*D-R	AACTGCAGCAAGACCGCAAGGAATTAATCG	*Pst*I
*lpxM*U-F	CCCAAGCTTGCGTTACGGCGATCATCACCAT	*Hind*III
*lpxM*U-R	CCCGGCTAGCAAAGATCTTTATCCCATCAAAT	*Nhe*I
*lpxM*D-F	GTGGATCCATCAAACTCAGGAATGTATTCGC	*BamH*I
*lpxM*D-R	ATGAGCTCATGGTCGCAGCTACACCACGCG	*Sac*I
*kan*-loxP-F	ATGAATTCGCGGCCGCATAACTTCGTATAA	*EcoR*I
*kan*-loxP-R	AGAGATCTGCGGCCGCATAACTTCGTATAA	*Bgl*II
loxpLE-F	TTACCGTTCGTATAATGTATGCTATACGAAGTTATA	/
loxpLE-R	TCGATATAACTTCGTATAGCATACATTATACGAACGGTAAGGCC	/
loxpRE-F	CTAGTATAACTTCGTATAATGTATGCTATACGAACGGTATAGC	/
loxpRE-R	TATACCGTTCGTATAGCATACATTATACGAAGTTATA	/
*kan*-F	TAACTCGAGATTCACGCTGCCGCAAGCACTCAGG	*Xho*I
*kan*-R	AAGTCTAGACGAACCCCAGAGTCCCGCTCAGA	*Xba*I
*kan*-loxpLE-F	ATGGATCCAATACGACTCACTATAGGGCG	*BamH*I
*kan*-loxpRE-R	ACCTCTAGAGCGCAATTAACCCTCACTAAAG	*Xba*I
*cre*-F	CGAGCTCAGGAGGTTATAAAGGATGTCCAATTTACTG	*Sac*I
	ACCGTACACCA	
*cre*-R	TACCATGGTCTAATCGCCATCTTCCAGCAG	*Nco*I
*lacZ*-F	CCATTCAGGCTGCGCAACTGTT	/
*lacZ*-R	TTAATGCAGCTGGCACGACAGG	/

### 3.2. Construction of Plasmids and Bacterial Strains

All bacterial strains and plasmids used in this study are listed in Table 2. Bacteria were usually grown at 37 °C in LB media (10 g/L trypton, 5 g/L yeast extract, 10 g/L NaCl). When necessary, antibiotics were added with a final concentration of 100 μg/mL for ampicillin, 30 μg/mL for kanamycin and 30 μg/mL for chloramphenicol.

Four plasmids pBS-Ikan, pDTW202, pBS-Mkan and pKD-Cre were constructed in this study (Figure 2a). Plasmid pBS-Ikan was constructed by inserting a DNA fragment, *lacI*U-loxp-*kan*-loxp-*lacI*D, in the vector pBlueScript II SK+. Primers *lacI*U-F and *lacI*U-R were used to amplify the upstream fragment of *lacI*; primers *lacI*D-F and *lacI*D-R were used to amplify the downstream fragment of *lacI*; primers *kan*-loxP-F and *kan*-loxP-R were used to amplify the fragment loxp-*kan*-loxp from pMG2-*kan*^r^ [20]. Plasmid pDTW202 was constructed by inserting a fragment, loxpLE-*kan*-loxpRE, in the vector pBlueScript II SK+. Fragments loxpLE and loxpRE were obtained by the annealing of the primers loxpLE-F, loxpLE-R and loxpRE-F, loxpRE-R, respectively, according to the method of Arakawa [21], and the kanamycin resistance gene *kan* was PCR amplified from pK18mobsacB [22] with primers *kan*-F and *kan*-R. Plasmid pBS-Mkan was constructed by inserting a fragment, *lpxM*U-loxpLE-*kan*-loxpRE-*lpxM*D, in the vector pBlueScript II SK+. Primers *lpxM*U-F and *lpxM*U-R were used to amplify the upstream fragment of *lpxM*; primers *lpxM*D-F and *lpxM*D-R were used to amplify the downstream fragment of *lpxM*, and primers *kan*-loxpLE-F and *kan*-loxpRE-R were used to amplify the fragment loxpLE-*kan*-loxpRE from pDTW202. Plasmid pKD-Cre was constructed by replacing the λ Red genes with the gene *cre* in the vector pKD46 [23]. The gene *cre* was amplified from the vector pSH47 [24], using primers *cre*-F and *cre*-R, digested with SacI and NcoI and ligated with the vector pKD46, which is similarly digested. The gene *cre* was put under the arabinose-inducible ParaB promoter. The plasmid pKD-Cre has a temperature sensitive replicon.

**Table 2:** Bacterial strains and plasmids used in this study.

Strains or Plasmids	Description	Source
Strains		
W3110	Wild-type *E. coli*, F^−^, λ^−^	Laboratory strain
W3110/pKD46	W3110 transformed by pKD46	This work
HW000	W3110 Δ*lacI*	This work
HW001	W3110 Δ*lacIlacZ*::*lpxE*	This work
HW002	W3110 Δ*lacI*Δ*lpxMlacZ*::*lpxE*	This work
Plasmids		
pKD46	P_araB_γβ exo, Rep^ts^,Amp^R^	[23]
pMG2-*kan*^r^	loxP-*kan*-loxP, Amp^R^, Kan^R^	[20]
pK18mobsacB	Mob^+^, sacB, Kan^R^	[22]
pDTW202	loxPLE-*kan*-loxPRE, Amp^R^, Kan^R^	This work
pCP20	FLP^+^, λ cI857^+^, λp_R_Rep^ts^, Cam^R^, Amp^R^	[23]
pSH47	GAL1p-Cre-CYC1T, Amp^R^	[24]
pKD-Cre	P_araB_ cre, Rep^ts^, Amp^R^	This work
pBlueScript II SK+	Cloning vector, ColE1, *lacZ*, Amp^R^	Stratagene
pBS-Ikan	Plasmid for deleting *lacI* in *E. coli*	This work
pBS-Mkan	Plasmid for deleting *lpxM* in *E. coli*	This work
pWSK29-*FnlpxE*-*Fkan*	Plasmid for containing *lpxE*	[19]

Three *E. coli* mutants, HW000, HW001 and HW002, were constructed by deleting and/or integrating genes in the chromosome of *E. coli* (Figure 2b). Firstly, the DNA fragment *lacI*U-loxp-*kan*-loxp-*lacI*D was amplified from pBS-Ikan using primers *lacI*U-F and *lacI*D-R and transformed into *E. coli* W3110/pKD46. Under the help of Red enzymes expressed by pKD46, the *lacI* gene in the chromosome should be replaced by DNA fragment loxp-*kan*-loxp; the transformants were selected on LB agar plates containing kanamycin. The *kan* gene inserted in the chromosome was further eliminated by transforming pKD-Cre. Plasmid pKD-Cre expresses the Cre recombinase, which directly acts on the repeated loxp (Cre recognition target) sites flanking the *kan* gene and excises the *kan* gene, resulting in the *lacI* mutant HW000. Secondly, the DNA fragment *lacZ*U-*lpxE*-FRT-*kan*-FRT-*lacZ*D was amplified from pWSK29-*FnlpxE-Fkan* [19] using primers *lacZ*-F and *lacZ*-R and transformed into HW000/pKD46. Under the help of Red enzymes expressed by pKD46, the *lacZ* gene was broken by the inserted DNA fragment *lpxE*-FRT-*kan*-FRT. The *kan* gene was then eliminated by transforming pCP20. Plasmid pCP20 expresses the FLP recombinase, which acts on the directly repeated FRT (FLP recognition target) sites flanking the *kan* gene and excises the *kan* gene [23], resulting in the double mutant HW001. Thirdly, the DNA fragment *lpxM*U-loxpLE-*kan*-loxpRE-*lpxM*D was amplified from pBS-Mkan using primers *lpxM*U-F and *lpxM*D-R and transformed into HW001. Under the help of Red enzymes expressed by pKD46, the *lpxM* gene in the chromosome was replaced by the DNA fragment loxpLE-*kan*-loxpRE. The *kan* gene was then eliminated by transforming pKD-Cre, resulting in the triple mutant HW002. Since the short sequences loxp left by deleting *lacI,* loxpLR left by deleting *lpxM* and FRT left by inserting *lpxE* are different, they could not recombine with each other in the double or triple mutants.

### 3.3. Isolation and Analysis of Lipid A from *E. coli* Strains

Typically, 200 mL cultures were inoculated from overnight cultures and grown to an OD_600_ of 1.0. The cells were harvested by centrifugation at 8000 rpm for 20 min and washed with PBS. The final cell pellet was resuspended in a single phase Bligh-Dyer mixture [25] (76 mL) consisting of chloroform/methanol/water (1:2:0.8 v/v) and agitated by stirring for 1 h at room temperature. The cell debris was collected by centrifugation at 2000 rpm for 20 min, and the insoluble material was washed twice with the same solvent. The pellets were resuspended in 27 mL 12.5 mM sodium acetate (pH 4.5) by sonication and heated at 100 °C for 30 min to release lipid A from LPS. After cooling to room temperature, the suspensions were converted to two-phase Bligh-Dyer mixtures consisting of chloroform/methanol/water (2:2:1.8 v/v) by adding 30 mL chloroform and 30 mL methanol. The mixture was vigorously shaken to extract lipid A and centrifuged at 2000 rpm for 20 min. The lower phase containing lipid A was dried with a rotary evaporator and stored at −20 °C.

The dried lipid A was dissolved in chloroform/methanol (4:1 v/v) and spotted onto a silica gel 60 TLC plate. The plate was developed in the solvent of chloroform/methanol/water/ammonia (40:25:4:2 v/v/v/v). After drying and spraying with 10% sulfuric acid in ethanol, the separation of lipid A on the plate could be visualized by charring at 175 °C.

For ESI/MS analysis, dried lipid A was dissolved in chloroform/methanol (4:1 v/v) and subjected to ESI/MS in the negative ion mode. The mass spectra were acquired on a Waters SYNAPT Q-TOF mass spectrometer equipped with an ESI source. Data acquisition and analysis were performed using MassLynx V4.1 software [26].

### 3.4. Extraction and Purification of LPS

LPS were isolated from *E. coli* strains using the phenol-water procedure [27]. Wet *E. coli* cells (3 g) were suspended in water (10 mL) at 68 °C, and 90% aqueous phenol (10 mL) was added. The mixture was stirred for 1 h at 68 °C, then cooled to 4 °C and centrifuged at 4000 rpm for 20 min to separate the water and phenol phases. The water phase was collected, dialyzed against water and lyophilized. The crude LPS (0.5 g) was resuspended in 100 mM Tris-HCl (pH 7.5, 10 mL) and then by treating with DNase I, RNase A at the concentration of 100 and 50 μg/mL at 37 °C for 2 h, respectively. Then 100 μg/mL proteinase K at 37 °C for 2 h was performed. For further purification, 5 mL water-saturated phenol was added to the crude LPS sample. The mixture was centrifuged at 4000 rpm for 20 min. The water phase was collected, dialyzed and lyophilized. Phospholipid contaminants in the sample were removed by extraction with chloroform/methanol (2:1 v/v) [28]. Purified LPS product was lyophilized and stored at 4 °C [29].

### 3.5. LPS Stimulation of the Murine Macrophage Cell Line RAW264.7

*E. coli* O111:B4 LPS was purchased from Sigma-Aldrich. The murine macrophage cell line RAW264.7 was obtained from the cell bank of the Chinese Academy of Science (Shanghai, China). It was maintained at 37 °C with 5% CO_2_ in DMEM (Gibco BRL, USA) containing 10% FBS (Hyclone Logan, UT, USA), supplemented with penicillin (100 U/mL) and streptomycin (100 μg/mL). Cells were cultured at 10^5^/well in 96-well plates with 200 μL medium each well. After 18 h, the RAW264.7 cells were stimulated with increasing concentration of LPS (1, 10, 100 and 1000 ng/mL). 24 h later, culture supernatants were collected, centrifuged to remove the contaminates and stored at −80 °C for cytokine analysis. Concentrations TNF-α were determined using the enzyme-linked immunosorbent assay kit (eBioscience), according to the manufacturer’s instruction.

One-way analysis of variance was performed to determine the statistical significance of the differences between mean values for various experimental and control groups. Data were expressed as means ± standard deviation, and the experiments were performed in triplicate. The means were compared using the least significant difference test. *p* < 0.05 was considered a significant difference and *p* < 0.01 was considered extremely significant difference. All data were analyzed with SPSS Statistics 17.0.

## 4. Conclusions

MPLA is safe and effective in eliciting Th1 response to heterologous proteins in animal and human vaccines [8]; therefore, it is becoming an important target molecule for developing novel vaccine adjuvant. MPLA can be chemically synthesized, but the chemically synthesized MPLA has a slightly different structure, which might affect its recognition by TLR4/MD-2 [30]. MPLA can also be prepared by extracting the lipid A from *Salmonella Minnesota* R595 and then removing the 1-phosphate group chemically. This MPLA product was 0.1% as toxic as wild-type LPS without affecting the immune-stimulating activity when tested in pre-clinical rabbit pyrogenicity assays [31,32]. However, this method has limitations on the efficiency and quality of MPLA production, because *S. minnesota* makes multiple lipid A species, which are difficult to separate from each other [33].

However, *E. coli*, the most common bacterium used for gene engineering, synthesizes only one type of lipid A. Therefore, we constructed two *E. coli* strains, HW001 and HW002, that could produce MPLA, by deleting or constitutively expressing genes related to lipid A biosynthesis in the chromosome. The gene *lpxE* encoding a phosphatase LpxE was integrated in the chromosome of HW001. LpxE can remove the phosphate at the 1-position of lipid A and has been used to develop *Salmonella* vaccines [34]. HW002 was constructed by deleting the gene *lpxM* in the chromosome of HW001. The *lpxM* gene product is an important virulence factor in a murine model of *E. coli* pathogenicity [35], and *Salmonella lpxM* mutants produce pentaacylated lipid A with reduced affinity to TLR4, resulting in virulence attenuation [36]. As expected, HW001 only synthesizes MPLA, and HW002 only synthesizes P-MPLA. Lower levels of cytokines TNF-α were released when RAW264.7 cells were stimulated by HW001 LPS and HW002 LPS than by the wild-type W3110 LPS (Figure 6), suggesting HW001 LPS and HW002 LPS are good candidates for developing lipid A vaccine adjuvant. 

Heterologous genes are usually expressed in *E. coli* using plasmids as the vector, but plasmids are not stable in bacteria and usually need antibiotics as selection markers. The markerless chromosomal integration of *lpxE* in HW001 and HW002 make these strains ideal for industrial production of MPLA. Other than LpxE and LpxM, there are enzymes, such as PagL and PagP, which could also modify the structure of lipid A to change its interaction with TLR4/MD-2 [37]. 
